# Quantitation of cyclic nucleotides in mammalian cells and in human urine by high-performance liquid chromatography/mass spectrometry

**DOI:** 10.1186/1471-2210-11-S1-P12

**Published:** 2011-08-01

**Authors:** Heike Burhenne, Kim Tappe, Kerstin Beste, Ulrike Voigt, Corinna Spangler, Dimitrios Tsikas, Volkhard Kaever, Roland Seifert

**Affiliations:** 1 Institute of Pharmacology, Hannover Medical School, Germany; 2 Institute of Clinical Pharmacology, Hannover Medical School, Germany

## Background

The cyclic nucleotides adenosine 3’,5’-cyclic monophosphate (cAMP) and guanosine 3’,5’-cyclic monophosphate (cGMP) are well-known second messengers. They play an important role in signal transduction. They control numerous functions ranging from ion channel opening to regulation of gene expression.

In 1963, cAMP and cGMP were detected in rat urine. Indeed, this was the first proof of cGMP in biolocigal systems [1]. In 1984, Newton et al. demonstrated the possible presence of cytidine 3’,5’-cyclic monophosphate (cCMP) in various rat tissues [2] and, two years later, of uridine 3’,5’-cyclic monophosphate (cUMP) [3] by fast atom bombardment. Furthermore, cCMP was supposedly detected in urine of patients with acute leukemia [4,5] by radioimmuno assay. These findings point to a biological function of cCMP and cUMP. However, due to significant methodological problems, studies of the biological function of cCMP und cUMP were no longer continued.

Recently, we have developed a method based on high-performance liquid chromatography-coupled mass spectrometry (HPLC-MS/MS), which allows the simultaneous determination of all cyclic nucleotides (cNMPs). Using this highly sensitive method we were able to detect and quantify cAMP, cGMP and cCMP as well as cUMP in various mammalian cell lines. We have optimized this method for the quantitation of cyclic nucleotides in complex biolocigal matrices, like urine and organs.

## Methods

Nucleotide extraction of cells was performed by treating cells with a mixture of organic solvents and heating the samples at 98°C. After centrifugation the supernatant fluid was evaporated under a nitrogen stream and the residual pellet was resuspended in water. Detection and quantitation of cNMPs was achieved by an analytical method based on HPLC-MS/MS.

To avoid serious matrix effects due to the complex urine composition we enzymatically synthesized ^13^C^15^N-labeled internal standards for each cyclic nucleotide. Urine sample preparation was achieved by treating urine with acetonitrile containing those standards. The read-out was performed as described above. The cCMP concentration was normalized to mmol creatinine. Creatinine concentration in urine was determined by gas chromatography -MS.

## Results and discussion

We could show that, in addition to cAMP and cGMP, cCMP and cUMP are present in all studied cell lines (Table 1). Remarkably, the cCMP- and cUMP-contents in HeLa cells are comparable to the cGMP contents, and in B103 cells the the cCMP- and cUMP-content is even two-fold and three-fold higher, respectively.

**Table 1:** 

*cNMP content* (*pmol/10^6^ cells*)									
*Cell type*	*Lineage*	*cAMP*		*cGMP*		*cCMP*		*cUMP*	
HeLA	epithelial cells	2.1	± 0.5	1.5	± 0.5	0.3	± 0.1	0.7	± 0.3
HL-60	myeloid cells	7.0	± 1.4	0.3	± 0.1	0.1	± 0.03	0.3	± 0.03
J774	macrophages	11.0	± 1.6	1.2	± 0.1	0.5	± 0.02	1.2	± 0.1
CHO	epithelial cells	33.3	± 4.0	10.2	± 1.3	8.0	± 1.6	6.7	± 1.8
B103	neuroblastoma cells	55.1	± 7.3	7.5	± 0.8	14.4	± 1.5	22.3	± 2.8
COS-7	fibroblasts	82.2	± 11.3	52.1	± 3.1	16.3	± 1.7	25.1	± 2.2
HEK 293	epithelial cells	106.5	± 11.3	73.3	± 5.2	32.6	± 6.2	54.9	± 11.3

Moreover, we analyzed human urine from healthy volunteers for cyclic nucleotides by HPLC-MS/MS. Our studies revealed that besides cAMP and cGMP, cCMP is present in human urine.

## Conclusion

Our data suggest that cCMP and cUMP play different roles in the regulation of the function of cell types originating from various cell lineages and species. The unequivocal identification of cCMP in human urine opens the door for (patho)physiological studies.
